# Significant Reduction in Peak Left Ventricular Global Longitudinal Strain in Multiple Myeloma Patients: A Pilot Random Awareness Analysis

**DOI:** 10.7759/cureus.80509

**Published:** 2025-03-13

**Authors:** Khalid Sawalha, Andrew J Fancher, Srikanth Vallurupalli, Angel Lopez Candales

**Affiliations:** 1 Cardiometabolic Medicine, University of Missouri Kansas City School of Medicine, Kansas City, USA; 2 Internal Medicine, University of Kansas School of Medicine–Wichita, Wichita, USA; 3 Cardiovascular Disease, University of Arkansas for Medical Sciences, Little Rock, USA; 4 Cardiovascular Medicine, University of Puerto Rico School of Medicine, San Juan, USA

**Keywords:** doppler echocardiography, left ventricular function, multiple myeloma, peak global longitudinal strain, right ventricular function

## Abstract

Background and methods

Multiple myeloma (MM) is a malignancy associated with cardiovascular complications. Though echocardiography plays a crucial role in assessing cardiac function, its use in the follow-up of MM patients is limited. For this descriptive analysis, we report several recommended objective measures of left ventricular (LV) systolic function, peak global longitudinal strain (PGLS), and right ventricular (RV) systolic function in 147 MM patients at various stages of their treatment who were referred to our echocardiography laboratory at the University of Arkansas for Medical Sciences (Little Rock, AR, USA).

Results

Mean age was 61 ± 11 years, (range: 30 to 86 years; 82 males). Mean left ventricular ejection fraction (LVEF) was 60 ± 9%, mitral annular (MA) systolic tissue Doppler velocity was 8 ± 2 cm/s, and LV outflow tract velocity time integral (LVOT VTI) was 21 ± 5 cm. Furthermore, RV systolic function measured by tricuspid annular plane systolic excursion (TAPSE) was 2.3 ± 0.4 cm and tricuspid annular (TA) tissue Doppler (TDI) systolic velocity (s') was 13 ± 3 cm/s. These values were reported with regards to normal published standard values. Finally, we identified three distinctive LV PGLS levels, ranging from -7% to -27%. Given the poor correlation, we identified that a total of 74% or 50% of all MM patients with normal LVEF (>55%) had abnormal LV PGLS. No correlation between LVEF and LV PGLS was seen in our study.

Conclusions

To our knowledge, this is the first study describing these objective measures of cardiac function in MM patients at different stages of treatment. However, additional studies are needed to truly assess asymptomatic changes in cardiac structure during MM treatment.

## Introduction

Despite being a relatively rare cancer (accounting for only 1% of all cancers), multiple myeloma (MM) ranks as the second most prevalent hematologic malignancy, surpassed only by lymphoma [1]. This condition encompasses a broad range of clinical presentations, spanning plasma cell dyscrasias to extramedullary myeloma, and is characterized by multiple relapses [2]. While predominantly affecting men slightly more than women, it is twice as common in African Americans compared to Caucasians with a median age at the time of diagnosis of about 65 years [3,4].

Better understanding of the underlying pathophysiology coupled with the recent introduction of novel drugs with improved efficacy, has significantly improved outcomes [5]. However, its onset at an older age, coupled with longer survival rates, places MM patients at a higher risk of developing heart disease and cardiovascular disease (CVD) mortality [6]. As a result of these associations, MM is now the third-most common type of malignancy associated with CVD [7].

From a CVD perspective, current literature suggests that MM patients are at risk for developing heart failure, uncontrolled systemic hypertension, accelerated ischemic heart disease, arterial/venous thromboembolism, and arrhythmias [8]. Specifically, left ventricular (LV) dysfunction in MM can occur due to increasing age, existing CVD risk factors, direct accumulation of amyloid light-chain immunoglobulins, toxicities from currently used agents, or secondary abnormalities caused by MM such as anemia, hypertension, drug-induced diabetes, as well as the development of chronic kidney disease [8].

Although this research study was commenced right before the rapid rise of COVID-19 cases in 2019, it is necessary to highlight that the widespread restrictions and limitations mandated at that time by health officials, precluded our ability to recruit more patients and obtain more valuable information of prior treatments. However, we believe that the data collected still provides valuable information regarding comprehensive echocardiographic assessment of cardiac function, including speckle tracking data, among a group of MM patients undergoing routine transthoracic studies at various stages of their treatment. We believe this data is particularly valuable, given the paucity of data on cardiac structural characteristics during the unrelenting course of MM.

## Materials and methods

For this retrospective, observational study, we reviewed our echocardiographic database echocardiograms conducted between August 2019 and February 2020 on MM patients referred to our echocardiography laboratory at the University of Arkansas for Medical Sciences (Little Rock, AR, USA). To be included into the final analysis all MM echocardiograms had to be complete, including our routinely acquired objective measures of LV systolic and diastolic function, as well as right ventricular (RV) systolic function. In addition, we also routinely collected LV peak global longitudinal strain (PGLS) unless two-dimensional image quality precluded accurate endocardial tracking, even after manual correction.

Echocardiographic studies were otherwise excluded if patients were not in sinus rhythm, ectopy was present, and thick papillary muscles or presence of pulmonary hypertension with distortion of the LV cavity was observed. Additionally, studies with inadequate orthogonal views or pericardial effusion larger than mild in size were excluded. Finally, none of the studies were either limited or performed by cardiology trainees.

Two-dimensional transthoracic echocardiography (TTE) studies were performed using commercially available systems (Vivid 9; GE Medical Systems, Milwaukee, WI,l USA). Images were obtained in the left lateral decubitus position with the patient in the supine position using a 3.5-MHz transducer. Standard two-dimensional, color, pulsed, and continuous-wave Doppler data were digitally acquired in gently held end-expiration and saved in regular cine loop format for subsequent offline analysis (EchoPAC version 111.0.00 and GE-Vingmed Ultrasound AS; GE Healthcare, Chicago, IL, USA).

The following echocardiographic and Doppler measures were obtained in all patients included in the final analysis. The LV ejection fraction (LVEF) was calculated using the Simpson's method as recommended by the American Society of Echocardiography, utilizing two-dimensional echocardiographic images to maximize areas while avoiding apical foreshortening [9]. In addition, a surrogate measure of LV systolic function used in this study was mitral annular (MA) systolic velocity (s') obtained from the lateral MA, as previously described [9]. We also utilized the LV outflow tract (LVOT) velocity time integral (VTI). The LVOT VTI measurement was obtained by placing the pulsed Doppler sample volume in the LVOT below the aortic valve and recording the velocity measured in cm/s, which correlates well with cardiac output [9].

Myocardial performance index (MPI), first described by Tei et al., was calculated by dividing the total duration of ejection by the interval denoted by isovolumic contraction time and isovolumic relaxation time using the tissue Doppler imaging signals for both left ventricle and right ventricles, as previously described [10]. Measures of LV diastolic function were determined using the 2016 recommended guidelines published by the American Society of Echocardiography [11].

**Figure 1:**
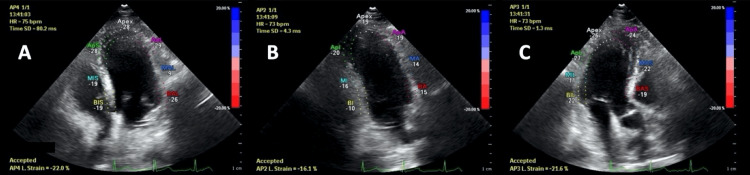
The four-chamber (A), two-chamber (B), and apical long axis (C) views showing the six points of AFI for speckle tracking interrogation of the LV cavity. AFI: Automated functional imaging, LV: Left ventricular

For speckle tracking imaging we measured LV PGLS using the Vivid 9's automated functional imaging (AFI) method to analyze speckle tracking. This echocardiographic imaging modality is useful when assessing LV longitudinal systolic function [12]. The three-click method was used to minimize variability and LV speckle tracking echocardiography (STE) data is acquired from endocardial tracings obtained from two- and four-apical chamber as well as apical long axis views with frame rates > 60 frames/sec (Figure 1). Once the tracking process is verified, the image is accepted. Data was collected and saved into the program to automatically trigger PGLS analysis [12].

In terms of RV echocardiographic and Doppler variables, we determined RV systolic function using both tricuspid annular plane systolic excursion (TAPSE) and tricuspid annular tissue Doppler systolic velocity (TA TDI s') [13]. The TAPSE was measured by total excursion from its highest position after atrial ascent to its maximal descent during ventricular systole, with the M-mode cursor placed on the lateral tricuspid annulus (TA) from the apical four-chamber [13]. Similarly, TA TDI s’ measurements were obtained using tissue Doppler imaging with the same four-chamber apical anatomical orientation as used with M-mode from the lateral TA, as previously described [13].

Maximal tricuspid regurgitation systolic velocity value was measured from multiple windows using continuous wave Doppler, and the highest velocity was used to estimate pulmonary artery systolic pressures using the modified Bernoulli equation [13]. An estimate of the mean right atrial pressure was then used to make the final calculation of pulmonary artery systolic pressures by determining the diameter and collapse index of the inferior vena cava with inspiration [13].

Pulsed rather than continuous wave Doppler to accurately measure the duration of the signal for acceleration and RV outflow tract velocity time integral (VTI) calculations, as previously described was also collected [14]. For this measurement, a 1 mm to 2 mm pulsed wave Doppler sample volume was located just within the pulmonary valve from the parasternal short-axis view [14]. The sample volume was placed so that the closing but not opening click of the pulmonary valve was visualized [14]. Finally, calculation of the TAPSE/pulmonary arterial systolic pressure (PASP) ratio was then performed, as described elsewhere [15].

The above-mentioned echocardiographic approach, allowed us to gather valuable echocardiographic data with respect to MM patients at different stages of their treatment. All continuous data are presented as mean ± SD. Categorical data are presented as frequencies or percentages. Correlations between LV PGLS measurements and conventional echocardiographic parameters were performed using Pearson’s correlation. Multivariate logistic regression analysis was performed to detect which of the measured echocardiographic measures best correlated. A p-value of <0.05 was considered statistically significant. All analyses were performed using SPSS Statistics version 17.0 (SPSS, Inc., Chicago, IL, USA).

## Results

In this descriptive analysis, a comprehensive set of echocardiographic and Doppler data comprising a total of seven measures for LV systolic and diastolic function, as well as seven measures for RV systolic function, were initially identified in a total of 167 consecutive echocardiograms from MM patients performed between August 2019 and February 2020 at our Echocardiography laboratory at the University of Arkansas for Medical Sciences. However, due to limited endocardial border resolution for acquisition of accurate LV PGLS data, 20 studies were excluded and the studied population was finally comprised of 147 MM patients with an average age of 61 ± 11 years, ranging from 30 to 86 years with 82 patients being male. Demographic information is displayed in Table 1. 

**Table 1:** Demographic information

Parameters	N
Mean age ± SD (years)	61 ± 11
Male patients (%)	82 (55.8%)

It is important to highlight the fact that none of the MM patients included in this study have had recent or active COVID infection at the time of the echocardiographic study. Measures of LV systolic and diastolic function were as follows: mean LVEF was 60 ± 9, with a range of 25 to 89; mitral valve E-velocity (MV E) was 78 ± 24 cm/s (range 38-168); mitral annulus e’ velocity (MA e’) was 9 ± 3 (range 3 -15); mitral annulus early diastolic velocity (E/e’ ratio) was 10 ± 5 (range 4-40); mitral annulus velocity (MA s’) was 8 ± 2 cm/s (range of 5 -1); LV myocardial performance index (MPI) was 0.49 ± 0.15 (range 0.09-0.93); and LVOT VTI was 21 ± 5 cm (range of 10 - 35) as displayed in Table 2. 

**Table 2:** The LV systolic and diastolic echo-Doppler data for a total of 147 MM patient LV: Left ventricular, MM: Multiple myeloma, LVEF: Left ventricular ejection fraction, MV E: Mitral valve E-velocity, MA e’: Mitral annulus e’ velocity, E/e’: Early diastolic velocity, MA s’: Mitral annulus velocity, MPI:  Myocardial performance index, LVOT VTI: Left ventricular outflow tract velocity time integral

Variables	Mean	Range	Normal value
LVEF (%)	60 ± 9	25-89	52%-72%
MV E (cm/s)	78 ± 24	38-168	50-100
MA e’ (cm/s)	9 ± 3	3-15	> 8
E/e’ ratio	10 ± 5	4-40	< 14
MA s’ (cm/s)	8 ± 2	5-15	> 6
LV MPI	0.49 ± 0.15	0.09-0.93	< 0.40
LVOT VTI (cm)	21 ± 5	10-35	20-30

**Figure 2:**
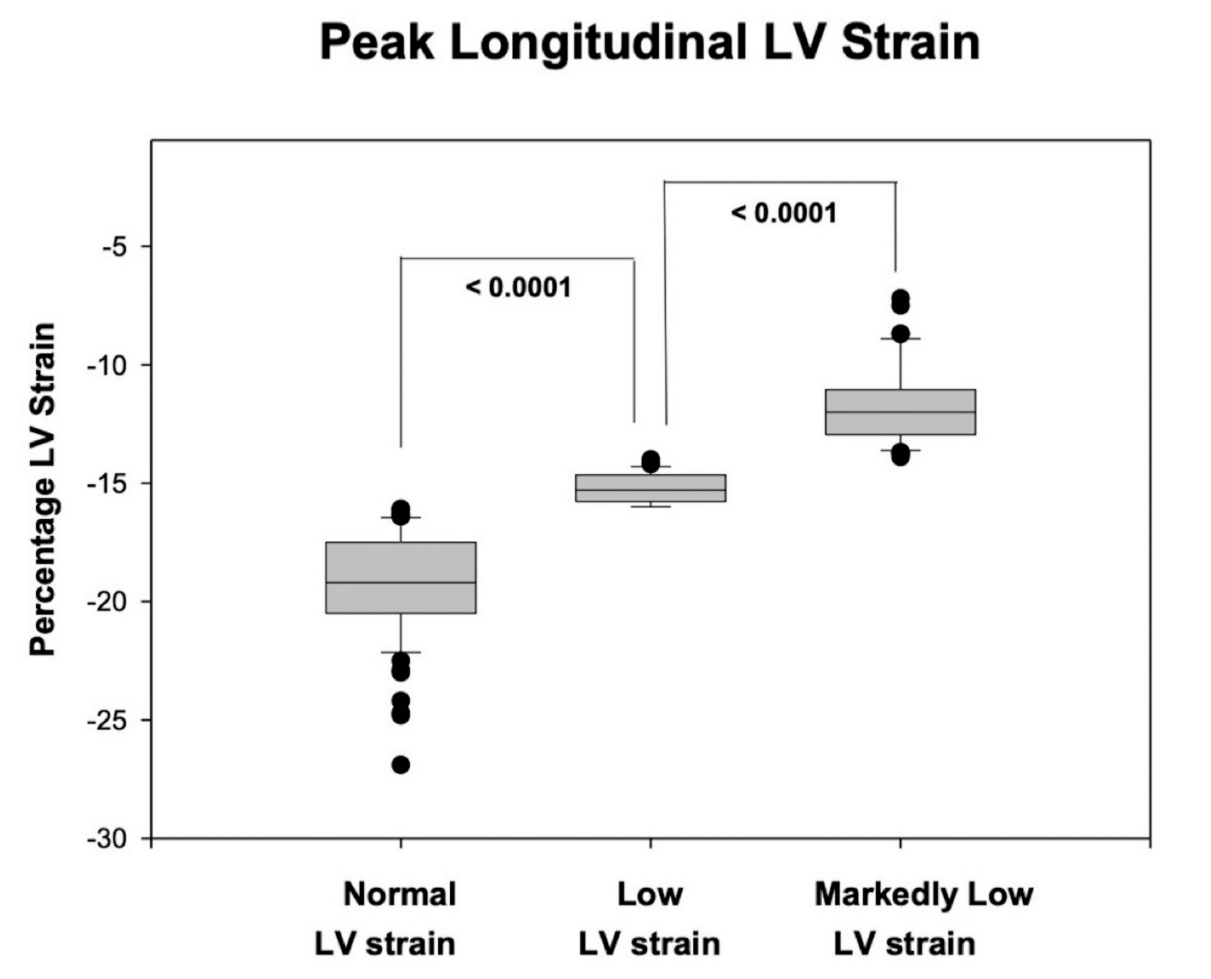
Box plot showing distribution of LV PGLS in our studied population during different stages of their treatment As seen, a total of 69 (47%) MM patients had a normal LV PGLS (-19.2 ± 2.3%, range of -16 to -27%). In contrast, 78 (53%) MM patients had an abnormal LV PGLS value. Of these abnormal values, further analysis disclosed that 25 (17%) MM patients had abnormally low normal LV PGLS levels (-15.2 ± 0.6%, range of -14 to -16%; p <0.0001) while 53 (38%) MM patients had markedly low LV PGLS values (-11.7 ± 1.7%, range -7 to -13%; p <0.0001). Correlations between LV PGLS measurements were performed using Pearson’s correlation. LV PGLS: Left ventricular peak global longitudinal strain, MM: Multiple myeloma

In our studied population LV PGLS was -16 ± 4% (range -7% to -27%); LVEF was 61 ± 8 (range 30% to 89%); mitral annular systolic velocity was 8.6 ± 2.2 (range 5 cm/s to 15 cm/s); MPI was 0.51 ± 0.17 (range 0.09-1.41) and LVOT VTI was 21 ± 5 (range 10-35 cm). Distribution of LV PGLS in our population is shown in Figure 2. 

**Figure 3:**
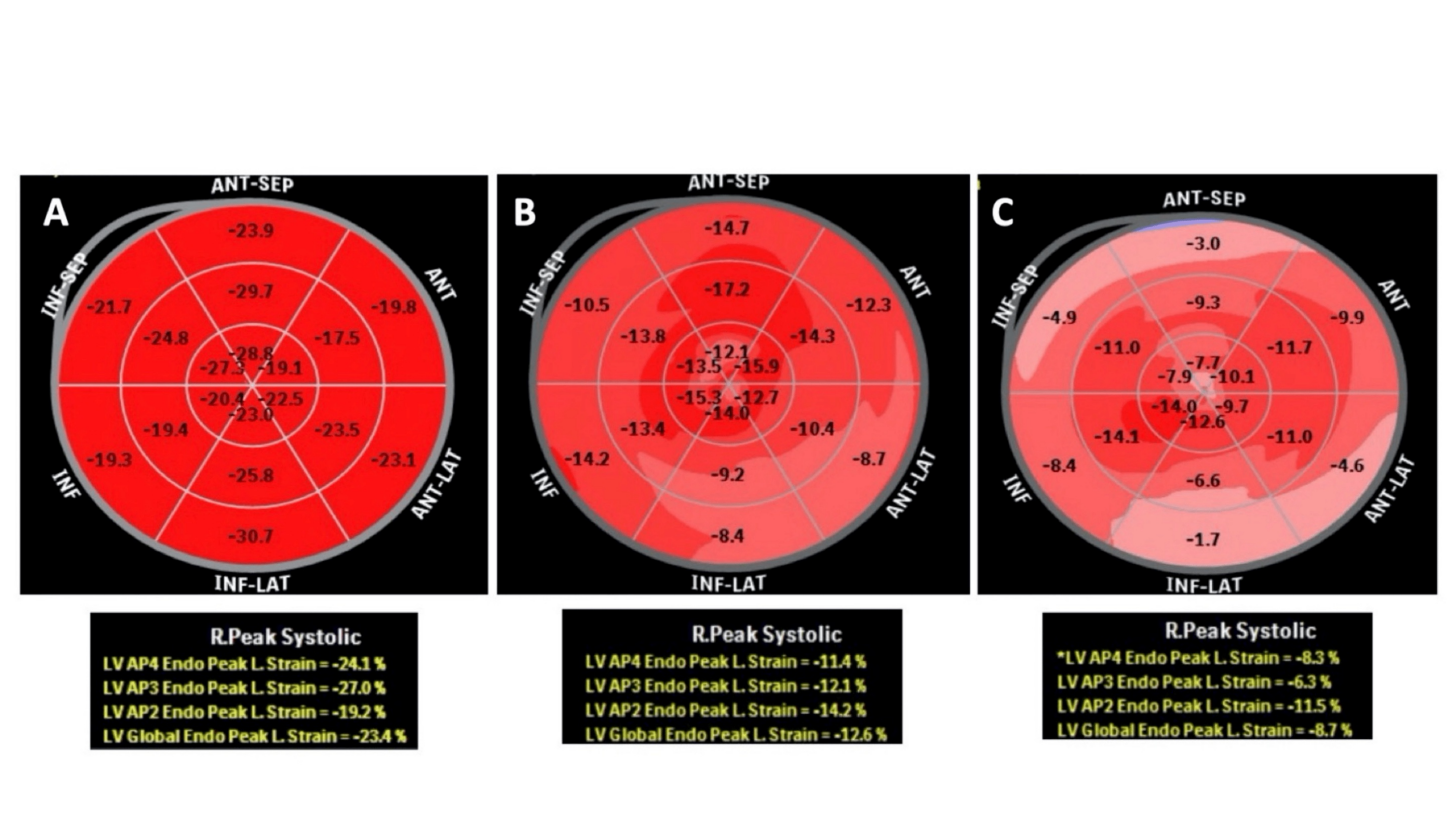
Representative LV PGLS polar maps derived from speckle tracking imaging algorithms using Vivid 9's AFI (GE Medical Systems) The polar display shows all myocardial wall segments, and the three inner circles represent LV basal, mid cavity and apical segments, respectively. The individual values represent strain measurements for each segment encoded in the LV image acquisition. The legend box below shows mean values for the LV four-apical chamber, three-chamber, and two-chamber image analysis. A final composite value from all three LV views is then reported as LV PGLS, which is the value of interest. The images shown in this figure are representative of the three LV PGLS groups featured in Figure 2, namely (A) MM patients with normal LV PGLS (-19.2 ± 2.3%, range of -16% to -27%) values; (B) MM patients with abnormal LV PGLS levels (-15.2 ± 0.6%, range of -14% to -16%; p <0.0001); and (C) MM patients with markedly low LV PGLS values (-11.7 ± 1.7%, range -7% to -13%; p <0.0001). LV PGLS: Left ventricular peak global longitudinal strain, MM: Multiple myeloma, AFI: Automated functional imaging

The LV PGLS was not obtained in 20 (12%) MM patients due to poor quality image. Careful analysis showed that a total of 69 (47%) MM patients had a normal LV PGLS (-19.2 ± 2.3%, range of -16 to -27%) as shown in Figure 2. In contrast, a total of 78 (53%) MM patients had an abnormal LV PGLS value. Of these abnormal values, further analysis disclosed that 25 (17%) MM patients had abnormally low normal LV PGLS levels (-15.2 ± 0.6%, range of -14 to -16%; p <0.0001) while 53 (38%) MM patients had markedly low LV PGLS values (-11.7 ± 1.7%, range -7 to -13%; p <0.0001) (Figure 2) values [15-18]. Representative LV PGLS polar maps from each of these three LV PGLS groups featured in Figure 2, can be shown in Figure 3.

**Figure 4:**
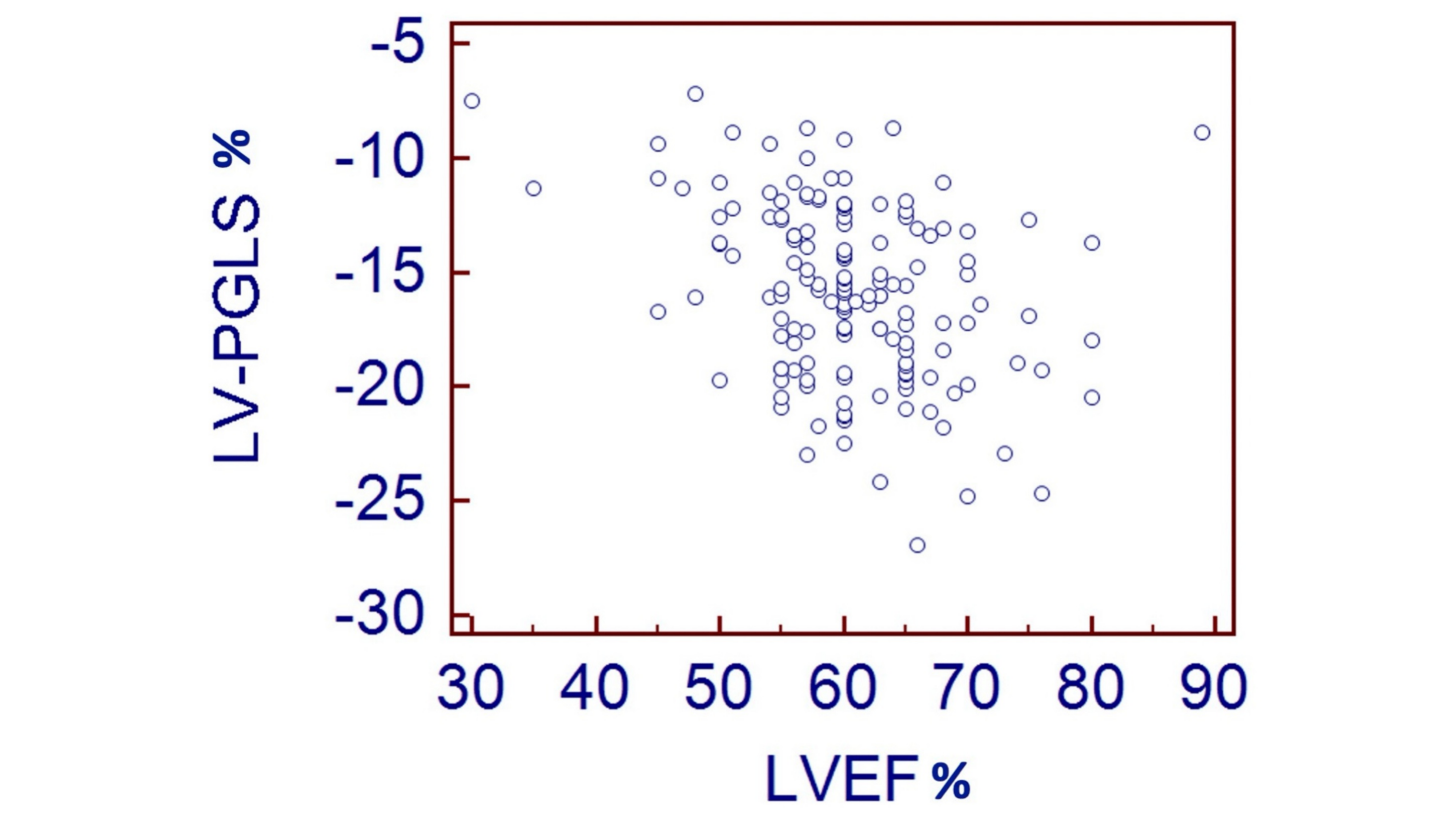
Correlation plot between LVEF and LV PGLS, both denoted as percentages, show no correlation in our MM studied population. LVEF: Left ventricular ejection fraction, LV PGLS: Left ventricular peak global longitudinal strain

Given the poor correlation known to exist between LV PGLS and LVEF, we examined our studied population and identified that a total of 74 (50%) MM patients with normal LVEF (>55%) had abnormal LV PGLS. No correlation between LVEF and LV PGLS was seen in our study as shown in Figure 4 [18].

Finally, when we performed a multiple regression analysis on our MM patient cohort (Table 3). The mitral annular systolic velocity correlated best with LV PGLS, followed by LVOT VTI and LVEF.

**Table 3:** Multiple regression analysis performed on the total cohort of 147 MM patients with complete echocardiographic data showed that mitral annular systolic velocity was the best parameter correlating with peak LV PGLS, followed by LVOT VTI and LVEF LV: Left ventricular, PGLS: Peak global longitudinal strain, MM: Multiple myeloma, LVOT VTI: Left ventricular outflow tract velocity time integral, MPI: Myocardial performance index, ICT: Isovolumic contraction time, IRT: Isovolumic relaxation time

Independent variables	Coefficient	Standard error	r_partial_	t	p-value
LVEF (%)	-0.1013	0.04157	-0.2067	-2.437	0.0161
Mitral annular systolic velocity (cm/s)	-0.6625	0.1420	-0.3750	-4.666	<0.0001
LVOT VTI (cm)	-0.2479	0.07567	-0.2732	-3.276	0.0013
MPI-ICT (ms)	-0.07632	0.06817	-0.09662	-1.120	0.2649
MPI-IRT (ms)	0.03845	0.04542	0.07320	0.846	0.3988
LV MPI	-14.4080	12.1250	-0.1025	-1.188	0.2368

Regarding RV systolic function, mean measurements were obtained for the following variables: TA TDI s' was 13 ± 3 cm/s (range 5-27), TAPSE was 2.3 ± 0.4 cm (range 1.4 to 3.6), and RV systolic pressure (RVSP) was 17 ± 12 mmHg (range of 5 to 51). The TAPSI/RVSP ratio ranged from 0.03 to 0.60, with a mean of 0.24 ± 0.18. The RV outflow tract acceleration time (RVOT acc) was 121 ± 23 msec (range 70 to 171), the RV VTI was 14 ± 4 cm (range 2 to 24), and the RV MPI was 0.45 ± 0.13 (0.23 to 0.95) as shown in Table 4.

**Table 4:** The RV systolic echo-Doppler data of 147 MM patients RV: Right ventricular, MM: Multiple myeloma, TA TDI s': Tricuspid annular tissue Doppler systolic velocity, TAPSE: Tricuspid annular plane systolic excursion, RVSP: Right ventricular systolic pressure, RVOT acc: Right ventricular outflow tract acceleration time, VTI: Velocity time integral, MPI: Myocardial performance index

Variables	Mean	Range	Normal value
TA TDI s' (cm/s)	13 ± 3	5-27	> 10
TAPSE (cm)	2.3 ± 0.4	1.4-3.6	> 1.6
RVSP (mmHg)	17 ± 12	5-51	< 25
TAPSE/RVSP ratio	0.24-0.18	0.03-0.60	> 0.36
RVOT acc (msec)	121 ± 23	70-171	-
RV VTI (cm)	14 ± 4	2-24	-
RV MPI	0.45 ± 0.13	0.23-0.95	< 0.40

## Discussion

The current study illustrates the wide range of cardiac function parameters in MM patients referred for routine echocardiographic studies. While the mean measures are normal, presence of both RV and LV structural abnormalities were common. It is now well recognized that the presence of either pre-existing or secondary cardiac effects of MM and novel treatment strategies are increasingly important when treating these patients [7,8,19,20]. Furthermore, by performing speckle tracking we were able to identify a wide range of clinically silent unrecognized abnormalities with regards to LV PGLS values. The latter data aligns with previous data recognizing the role of STE role in early detection of subclinical cardiac dysfunction, given the poor correlation existing between LV PGLS and LVEF [16-18].

Despite continuous improvement in survival of MM patients, true estimates may be overestimated since current data is mainly derived from randomized controlled trials in which patients with poor performance status and significant comorbidities were excluded. Furthermore, relatively low rates of patient enrollment in clinical trials in the US, and inadequate representation of different patient subgroups hinders understanding of the wide range of patient cardiac risk profiles [21].

Our study indicates, as described by others, that a significant heterogeneity exists regarding cardiac functional abnormalities which at times remain clinically silent and may affect a patient's clinical or treatment response over time. This study included a heterogeneous group of patients during different stages of treatment for whom we had no information regarding tumor burden, presence of cytogenetic abnormalities, treatments received, and overall response to therapy.

Although cardiotoxicity associated with adriamycin was initially shown in the 1970s [22], the ongoing development of a whole host of novel targeted therapies has increased the number of agents that are now available and potentially cardiotoxic. This prompted the development of the field of cardio-oncology to provide early detection, monitoring, and institution of appropriate protective cardiovascular therapies to mitigate the toxicity related to anticancer therapy [23]. In the case of MM, modern therapies, particularly proteasome inhibitors, bortezomib, and carfilzomib are known to be cardiotoxic, causing heart failure [24]. Furthermore, the development of cardiac amyloidosis is another detrimental complication of MM that compromises the survival of MM cases complicated by light-chain amyloidosis [24]. Therefore, detection of pre-existing LV and RV abnormalities is invaluable to identify patients at risk for potential treatment complications and prompt the implementation of preemptive cardioprotective treatments. Coupled with these approaches, regular cardiovascular monitoring should play a key role in detecting early cardiotoxicity in MM survivors. 

Even though routine monitoring methods are currently in place regarding periodic clinical assessment among MM patients, our data suggests that assessment of cardiac performance, using echocardiography, should be considered part of these assessments. The latter might assist oncologists in modifying or interrupting potentially cardiotoxic therapies and make appropriate referrals for cardioprotective medications (e.g., beta-blockers, angiotensin-converting enzyme inhibitors) [8,24]. These interventions aim to preserve cardiac function, reduce the risk of cardiac events, and improve the long-term prognosis of MM survivors [8].

Despite the availability of several imaging techniques such as radionuclide nuclear medicine, cardiac MRI, and advanced echocardiography as potential noninvasive imaging tools for the overall assessment of cardiotoxicity, [25-27] the increased healthcare costs have driven providers to identify which imaging modality provides the most useful information cost-effectively. To that end and given the paucity of data regarding these echocardiographic parameters among MM patients, we describe the potential utility of objective echocardiographic measures as suggested by the American Society of Echocardiography [9,11,13] as well as speckle tracking imaging [16-18] in patients with MM during different stages of their treatment. Aside from the ready availability of transthoracic Doppler echocardiograms, this noninvasive imaging tool provides prompt and reproducible assessments of cardiac function that allows clinicians to guide treatment, optimize supportive care strategies, as well as facilitate risk stratification and prognostication of high-risk patients [9,11,13,28].

To our knowledge, this is the first study to describe several LV and RV echo-Doppler variables as well as LV PGLS values in MM patients at different stages of treatment. However, we acknowledge the following limitations. First, the small sample size, a common limitation of cancer-based studies. Nevertheless, this is the largest dataset on MM patients to our knowledge. Since this is a descriptive study, it serves as the foundation from which larger prospective studies could incorporate routine echocardiographic-Doppler measurements from the time of diagnosis throughout different treatment sessions to determine how these variables would be useful in managing MM patients over time, particularly as they develop dyspnea and fatigue. Second, some might argue about the lack of specific details regarding the dates and times of bone marrow transplant, as well as prior chemotherapy and/or immunomodulatory treatments. However, as a referral center, most of the patients enrolled in our analysis had their first echocardiogram at our institution as part of their initial assessment and their outside information was not available upon collecting data.

As previously mentioned, our study was hampered by the unfortunate rise of COVID-19 cases right before we launched our echocardiographic study. The widespread restrictions and limitations, mandated by health officials during that time, precluded our ability to recruit more patients and obtain more valuable information on prior treatments. One thing is certain, none of the referred MM patients had any recent or ongoing COVID infection at the time of the echocardiographic study. Therefore, despite these limitations, we were able to conduct a descriptive analysis with the original purpose of assessing the variables typically used for LV and RV function assessment, not previously performed in MM patients.

With the recent introduction of numerous new therapies over the past five years, it is necessary to pursue these noninvasive echocardiographic assessments in complex cases particularly when new mutations continue to occur throughout treatment [29]. Future studies must not only increase MM patient sample size but also incorporate echocardiography using objective measures to follow up on patients with MM.

## Conclusions

Echocardiography plays a vital role in the assessment of cardiac structure and function in MM. Our study suggests that the evaluation of LV PGLS may provide valuable insight into subclinical cardiac dysfunction even when LV EF is normal. Our data suggests that echocardiography with speckle tracking imaging, a readily available, cost-effective, and noninvasive imaging modality, should be routinely incorporated into standard treatment protocols to detect early changes from disease progression as well as cardiotoxicity. More importantly, this parameter should be used in clinical trials to ensure that cardiotoxicity risk is stratified by baseline PGL. Future studies should incorporate clinical outcomes to determine the prognostic impact of longitudinal changes in echo Doppler parameters in managing patients with MM.

## References

[1] Siegel RL, Miller KD, Wagle NS, Jemal A (2023). Cancer statistics, 2023. CA Cancer J Clin.

[2] (2025). National Cancer Institute. Surveillance, Epidemiology and End-Results Program. Cancer Stat Fact Sheets: Myeloma. SEER Data. https://seer.cancer.gov/statfacts/html/mulmy.html.

[3] Landgren O, Weiss BM (2009). Patterns of monoclonal gammopathy of undetermined significance and multiple myeloma in various ethnic/racial groups: support for genetic factors in pathogenesis. Leukemia.

[4] Kyle RA, Gertz MA, Witzig TE (2003). Review of 1027 patients with newly diagnosed multiple myeloma. Mayo Clin Proc.

[5] Kazandjian D (2016). Multiple myeloma epidemiology and survival: a unique malignancy. Semin Oncol.

[6] Austin PC, Lee DS, Fine JP (2016). Introduction to the analysis of survival data in the presence of competing risks. Circulation.

[7] Al-Kindi SG, Oliveira GH (2016). Prevalence of preexisting cardiovascular disease in patients with different types of cancer: the unmet need for onco-cardiology. Mayo Clin Proc.

[8] Camilli M, La Vecchia G, Lillo R (2021). Cardiovascular involvement in patients affected by multiple myeloma: a comprehensive review of recent advances. Expert Rev Hematol.

[9] Lang RM, Badano LP, Mor-Avi V (2015). Recommendations for cardiac chamber quantification by echocardiography in adults: an update from the American Society of Echocardiography and the European Association of Cardiovascular Imaging. J Am Soc Echocardiogr.

[10] Tei C, Nishimura RA, Seward JB, Tajik AJ (1997). Noninvasive Doppler-derived myocardial performance index: correlation with simultaneous measurements of cardiac catheterization measurements. J Am Soc Echocardiogr.

[11] Nagueh SF, Smiseth OA, Appleton CP (2016). Recommendations for the evaluation of left ventricular diastolic function by echocardiography: an update from the American Society of Echocardiography and the European Association of Cardiovascular Imaging. J Am Soc Echocardiogr.

[12] Mor-Avi V, Lang RM, Badano LP (2011). Current and evolving echocardiographic techniques for the quantitative evaluation of cardiac mechanics: ASE/EAE consensus statement on methodology and indications endorsed by the Japanese Society of Echocardiography. J Am Soc Echocardiogr.

[13] Rudski LG, Lai WW, Afilalo J (2010). Guidelines for the echocardiographic assessment of the right heart in adults: a report from the American Society of Echocardiography endorsed by the European Association of Echocardiography, a registered branch of the European Society of Cardiology, and the Canadian Society of Echocardiography. J Am Soc Echocardiogr.

[14] López-Candales A, Shaver J, Edelman K, Candales MD (2012). Temporal differences in ejection between right and left ventricles in chronic pulmonary hypertension: a pulsed Doppler study. Int J Cardiovasc Imaging.

[15] Tello K, Axmann J, Ghofrani HA (2018). Relevance of the TAPSE/PASP ratio in pulmonary arterial hypertension. Int J Cardiol.

[16] Urheim S, Edvardsen T, Torp H, Angelsen B, Smiseth OA (2000). Myocardial strain by Doppler echocardiography. Validation of a new method to quantify regional myocardial function. Circulation.

[17] Yang H, Wright L, Negishi T, Negishi K, Liu J, Marwick TH (2018). Research to practice: assessment of left ventricular global longitudinal strain for surveillance of cancer chemotherapeutic-related cardiac dysfunction. JACC Cardiovasc Imaging.

[18] Karlsen S, Dahlslett T, Grenne B, Sjøli B, Smiseth O, Edvardsen T, Brunvand H (2019). Global longitudinal strain is a more reproducible measure of left ventricular function than ejection fraction regardless of echocardiographic training. Cardiovasc Ultrasound.

[19] Li W, Garcia D, Cornell RF (2017). Cardiovascular and thrombotic complications of novel multiple myeloma therapies: a review. JAMA Oncol.

[20] Patel VG, Cornell RF (2019). Cardiovascular complications associated with multiple myeloma therapies: incidence, pathophysiology, and management. Curr Oncol Rep.

[21] Gore L, Ivy SP, Balis FM (2017). Modernizing clinical trial eligibility: recommendations of the American Society of Clinical Oncology-Friends of Cancer Research minimum age working group. J Clin Oncol.

[22] Lefrak EA, Pitha J, Rosenheim S, Gottlieb JA (1973). A clinicopathologic analysis of adriamycin cardiotoxicity. Cancer.

[23] Alvarez-Cardona JA, Ray J, Carver J (2020). Cardio-oncology education and training: JACC Council perspectives. J Am Coll Cardiol.

[24] Proskuriakova E, Jada K, Djossi SK, Khedr A, Neupane B, Mostafa JA (2021). Mechanisms and potential treatment options of heart failure in patients with multiple myeloma. Cureus.

[25] Löffler AI, Salerno M (2018). Cardiac MRI for the evaluation of oncologic cardiotoxicity. J Nucl Cardiol.

[26] Yu C, Pathan F, Tan TC, Negishi K (2021). The Utility of Advanced Cardiovascular Imaging in Cancer Patients-When, Why, How, and the Latest Developments. Front Cardiovasc Med.

[27] Berenguer DR, de Moraes Chaves Becker M, de Oliveira Buril R, Bertão PA, Filho BM, Brandão SC (2024). Progression of myocardial 18F-FDG uptake in a patient with cardiotoxicity. Arq Bras Cardiol.

[28] Mitchell C, Rahko PS, Blauwet LA (2019). Guidelines for performing a comprehensive transthoracic echocardiographic examination in adults: recommendations from the American Society of Echocardiography. J Am Soc Echocardiogr.

[29] Soloveva M, Solovev M, Risinskaya N (2024). Loss of heterozygosity and mutations in the RAS-ERK pathway genes in tumor cells of various loci in multiple myeloma. Int J Mol Sci.

